# Immunoregulatory effects triggered by immunobiotic *Lactobacillus jensenii* TL2937 strain involve efficient phagocytosis in porcine antigen presenting cells

**DOI:** 10.1186/s12865-016-0160-1

**Published:** 2016-06-24

**Authors:** Kohichiro Tsukida, Takuya Takahashi, Hikaru Iida, Paulraj Kanmani, Yoshihito Suda, Tomonori Nochi, Shuichi Ohwada, Hisashi Aso, Sou Ohkawara, Seiya Makino, Hiroshi Kano, Tadao Saito, Julio Villena, Haruki Kitazawa

**Affiliations:** Food and Feed Immunology Group, Laboratory of Animal Products Chemistry, Graduate School of Agricultural Science, Tohoku University, Sendai, Japan; Livestock Immunology Unit, International Education and Research Center for Food Agricultural Immunology (CFAI), Graduate School of Agricultural Science, Tohoku University, Sendai, Japan; Department of Food, Agriculture and Environment, Miyagi University, Sendai, Japan; Infection Immunology Unit, International Education and Research Center for Food Agricultural Immunology (CFAI), Graduate School of Agricultural Science, Tohoku University, Sendai, Japan; Cell Biology Laboratory, Graduate School of Agricultural Science, Tohoku University, Sendai, Japan; Agricultural & Veterinary Division, Meiji Seika Pharma Co., Ltd., Yokohama, Japan; Division of Research and Development, Meiji Co., Ltd., Odawara, Kanagawa Japan; Laboratory of Immunobiotechnology, Reference Centre for Lactobacilli (CERELA-CONICET), San Miguel de Tucumán, Tucuman Argentina

**Keywords:** *Lactobacillus jensenii*, Immunobiotics, Porcine antigen presenting cells, Blood monocytes-derived dendritic cells

## Abstract

**Background:**

Immunobiotic *Lactobacillus jensenii* TL2937 modulates porcine mononuclear phagocytes from Peyer’s patches (PPMPs) and induces a differential production of pro- and anti-inflammatory cytokines in response to Toll-like receptor (TLR)-4 activation. In view of the important role played by phagocytosis in the activation of antigen presenting cells (APCs), the aim of the present work was to examine the interaction of TL2937 with porcine PPMPs focusing on phagocytosis. In addition, this study aimed to investigate whether the effects of *L. jensenii* TL2937 in porcine blood monocyte-derived dendritic cells (MoDCs) are similar to those found in PPMPs considering that MoDCs do not recapitulate all functions of mucosal APCs.

**Results:**

Studies showed a high ability of porcine CD172a^+^ PPMPs to phagocytose *L. jensenii* TL2937. Interestingly, our results also revealed a reduced capacity of the non-immunomodulatory *L. plantarum* TL2766 to be phagocytosed by those immune cells. Phagocytosis of *L. jensenii* TL2937 by porcine PPMPs was partially dependent on TLR2. In addition, we demonstrated that TL2937 strain was able to improve the expression of IL-1β, IL-12 and IL-10 in immature MoDCs resembling the effect of this immunobiotic bacterium on PPMPs. Moreover, similarly to PPMPs those immunomodulatory effects were related to the higher capacity of TL2937 to be phagocytosed by immature MoDCs.

**Conclusions:**

Microbial recognition in APCs could be effectively mediated through ligand-receptor interactions that then mediate phagocytosis and signaling. For the immunobiotic strain TL2937, TLR2 has a partial role for its interaction with porcine APCs and it is necessary to investigate the role of other receptors. A challenge for future research will be advance in the full understanding of the molecular interactions of immunobiotic *L. jensenii* TL2937 with porcine APCs that will be crucial for the successful development of functional feeds for the porcine host. This study is a step in that direction.

## Background

In the past decades, there has been a great advance in the knowledge of professional phagocytes’ cellular and molecular interactions with microbes. Phagocytes, including macrophages, dendritic cells (DCs), and granulocytes, are key players in the sensing of the extracellular environment and therefore, they have a prominent role in the host response to microorganisms. Phagocytes are equipped with different cell receptors that have the ability to recognize and respond to pathogenic and innocuous microorganisms [1]. These receptors are called pattern recognition receptors (PRRs) and, are able to recognize ligands expressed on microorganisms known as microbial-associated molecular patterns (MAMPs). PRRs-MAMPs interaction is a key event in the generation of immune responses [1].

Phagocytosis of microorganisms by antigen presenting cells (APCs) involves actin remodeling and the reorganization of the membrane that allows the appropriate formation of the phagocytic vesicle. The grouping of phagocytic receptors and the concentration of other cofactors within the bilayer that favors PRRs-MAPMs interactions also characterize this process. Activation of PRRs by MAMPs induce signaling pathways and the final response of APCs is given by the different types of PRRs-MAPMs interactions produced [2].

As stated by Miaczynska et al. [3], endosomes/phagosomes are therefore not a simple storage compartment where phagocytosed microbes accumulate but instead constitute active signaling platforms. In this regard, some studies reported that signaling via Toll-like receptors (TLRs) modulates phagocytosis and that a complex interaction between phagocytosis and TLR signaling exists [4].

DCs form a heterogeneous group of APCs that are key players in the induction and regulation of immune responses, and therefore potential targets for therapeutic intervention in immune-related diseases [5]. Among DCs, intestinal DCs are central not only for maintaining protective immunity against pathogens but also preventing inflammatory intestinal immune responses against the microbiota and food antigens [6]. In response to the commensal microbiota, DCs contribute to tolerogenic immune responses through PRRs modulation [7]. In particular, several works demonstrated that TLR-dependent activation of DCs determine protection or immune tolerance that maintains immune homeostasis in the gut [7–9]. In relation to the induction of tolerance by TLRs, it was demonstrated that tolerogenic gut CD103^+^ DCs express thymic stromal lymphopoietin (TSLP) in response to TLR ligation in a MyD88-dependent manner, and that TSLP produced by DCs acts directly on T cells promoting the development of regulatory Foxp3^+^ T cells [10]. In addition, by generating mice that specifically lack DCs-expressed TRAF6, that is an adaptor protein able to integrate upstream signals from MyD88-dependent IL-1R-TLR superfamily pathways, Han et al. [11] were able to demonstrate a role for TRAF6 in directing DCs maintenance of intestinal immune tolerance.

Taking into consideration the capacity of intestinal DCs to promote tolerance in response to innocuous commensal microbes, researchers have use these cells to select bacterial strains with the ability to prevent or reduce the severity of intestinal inflammatory disorders [9]. These beneficial bacterial strains with immunomodulatory properties are called immunobiotics, and belong to the genera *Lactobacillus* and *Bifidobacterium*. Several studies have provided categorical evidence that the immunoregulatory mechanisms behind the beneficial effects of immunobiotics are related to their capacity to functionally modulate DCs activities [12–14].

Research work evaluating the effect of immunobiotics on APCs has been performed mainly in mice models or human cell lines, and few studies investigated those effects in livestock animals, such as pigs. In this regard, we demonstrated that the immunobiotic strain *Lactobacillus jensenii* TL2937 was able to modulate mononuclear phagocytes from porcine Peyer’s patches (PPMPs) that resulted in a differential cytokine profile in response to Gram negative bacteria or lipopolysaccharide (LPS) [15]. The immunomodulatory effect of *L. jensenii* TL2937 was related to an upregulation of the expression of three negative regulators of TLRs: single immunoglobulin IL-1-related receptor (SIGIRR), the ubiquitin-editing enzyme A20, and interleukin-1 receptor-associated kinase M (IRAK-M). Moreover, our previous work demonstrated that those effects were partially dependent on TLR2 activation [15]. In addition, we found that the use of *L. jensenii* TL2937 as a supplemental additive for piglets feeding could be a strategy to improve immune-health, growth performance and productivity in post-weaning pigs [16]. The *in vivo* experiments in pig showed not only the capacity of *L. jensenii* TL2937 strain to modulate mucosal immunity but to decrease plasma alternative complement activity and C reactive protein levels, indicating a beneficial effect in the systemic inflammatory status of pigs [16].

Considering the prominent role played by phagocytosis in the activation and modulation of APCs, the aim of this work was to examine the interaction of *L. jensenii* TL2937 with porcine PPMPs focused on phagocytosis. In addition, considering that MoDCs do not recapitulate all functions of mucosal APCs this study also aimed to investigate whether the effects of *L. jensenii* TL2937 in porcine blood monocytes and monocyte-derived dendritic cells (MoDCs) are similar to those observed in PPMPs. In our previous work [15], three different populations of APCs in swine PPs were defined using CD172a and CD11R1 as markers: CD172a^+^CD11R1^high^, CD172a-CD11R1^low^, and CD172a^+^CD11R1^−^ cells. We demonstrated that immunobiotic *L. jensenii* TL2937 induce a tolerogenic profile in APCs from porcine PPs expressing CD172a, and therefore we focused our studies in CD172a^+^ APCs populations in this work.

## Methods

### Microorganisms

Two *Lactobacillus* strains *L. jensenii* TL2937 and *L. plantarum* TL2766 were used in this study. Each strain was grown in Man-Rogosa-Sharpe (MRS) medium (Difco, Detroit, MI, USA) at 37 °C for 16 h. Bacteria were washed with PBS, and heat-killed (56 °C, 30 min). These bacterial samples were suspended in Dulbecco’s Modified Eagle Media (DMEM, Thermo Fisher Scientific Inc.), enumerated with a Petroff-Hausser counting chamber, and stored at −80 °C until use as described previously [15, 17].

### Obtainment of porcine Peyer’s patches mononuclear phagocytes (PPMPs)

All experimental procedures in animals were conducted in accordance with the Animal Experimentation Guidelines of Tohoku University (Sendai, Japan). Suspensions of porcine Peyer’s patches (PPs) immunocompetent cells were prepared from the ileum of adult swine according to our previous studies with some modifications [15, 18, 19]. Briefly, PPs were cut into fragments and then smoothly pressed through a nylon mesh, and washed with complete RPMI 1640 medium (Sigma, St Louis, MO) supplemented with 10 % FCS (Sigma). A hypotonic solution (0.2 % NaCl) was used to eliminate residual red cells and, a rescue was performed with an equal volume of a hypertonic solution (1.5 % NaCl). Finally, immune cells were fractionated using density gradient centrifugation (Lympholyte-Mammal, Cedarlane, Hornby, Ontario, Canada), and suspended in complete DMEM (Invitrogen, Tokyo, Japan) containing 10 % FCS (Sigma), 50 μg/ml streptomycin/penicillin, and 50 μg/ml gentamycine (Nacalai Tesque, Kyoto, Japan).

In order to isolate adherent mononuclear phagocytes, immune cells from PPs suspensions were placed into 2-well glass plates (Iwaki, Tokyo, Japan) in a concentration of 5 × 10^7^ cells/ml, and incubated for 2 h (37 °C and 5 % CO_2_ atmosphere) to allow cells to adhere to the glass surface. Subsequently, these glass plates were washed gently with complete RPMI 1640 medium (Sigma) to remove non-adherent cells [15]. Mononuclear phagocytes obtained by this protocol were referred as adherent PPMPs.

### Obtainment of porcine Peyer’s patches CD172a^+^ cells

CD172a^+^ cells were isolated from porcine PPs immune cells using a magnetically activated cell sorting (MACS) separation system (Miltenyi Biotec GmbH, Bergisch Gladbach, Germany) as described previously [15]. Briefly, cells were treated with anti-porcine CD172a-biotin-conjugated SWC3a IgG1 (Southern Biotech, Birmingham, AL), and streptavidin microbeads, and the 25 LS MACS separation columns were used. All procedures were conducted following the instructions of the manufacturer. This protocol allowed the obtainment of CD172a^+^ cells with purity higher than 96 % as demonstrated by flow cytometry analysis. Cells obtained by this protocol were referred as isolated CD172a^+^ PPMPs.

### Generation of porcine blood monocyte-derived dendritic cells (MoDCs)

Porcine blood monocyte-derived dendritic cells (MoDCs) were generated by a five-day protocol as previously described by [20]. Briefly, porcine monocytes were obtained from freshly collected porcine peripheral blood by a specific gravity centrifugal method with Lympholyte-mammal (CEDARLANE, HornBy, Ontario, Canada). Cells were cultured in complete RPMI1640 culture medium containing: 2 mM of l-glutamine, 100 U/ml of Polymixin B, 10 % of FCS, 100 ug/ml of penicillin, 100 U/ml of streptomycin, 20 ng/ml of recombinant porcine GM-CSF (R & D Systems), and 20 ng/ml of recombinant porcine IL-4 (R & D Systems). Cells were cultured at a density of 5.0 × 10^6^ cells/well (12-well plate), or 2.0 × 10^7^cells/well (6-well plate) at 37 °C in 5 % CO_2_ atmosphere. Cells were incubated for 5 days with cytokine-containing medium that was replaced on day 3. These cells are referred as immature MoDcs. For the obtainment of mature DCs, LPS (1 μg/ml, L2630 Sigma, lipopolysaccharides from *Escherichia coli* 0111:B4) was added to the medium on day 3. Mature MoDCs were harvested on day 5 or 7 using cell dissociation enzyme-free Hank’s-based buffer (Gibco® Cell Dissociation Buffer), and suspended in complete RPMI1640.

### Stimulation of porcine antigen presenting cells

Evaluation of the interaction of porcine APCs with lactobacilli was performed using *L. jensenii* TL2937, or *L. plantarum* TL2766 and PPMPs or MoDCs obtained as described above. In each case, cells were plated at a density of 1.5 × 10^6^ cells/well in 12-well type I collagen-coated or in 2-well glass plates. Lactobacilli were added to each well at a concentration of 5 × 10^8^ cells/ml, and cells were stimulated for 12 h. In addition, unlabeled anti-human TLR2 rabbit IgG (Santa Cruz, CA) was used in blocking experiments. Cultured cells were incubated with the unlabeled anti-human TLR2 antibodies for 12 h before stimulation with lactobacilli. In some experiments the TLR2 agonist Pam3CSK4 (200 ng/ml; EMC Microcollection, Tubingen, Germany) was used to stimulate cells.

### Immunofluorescence

Immunofluorescence analysis of porcine MoDCs was conducted as described in our previous reports [15]. Mounted MoDCs were incubated for 20 min with 1 % bovine serum albumin and 2 % normal goat serum (Vector Laboratories, Burlingame, CA) in PBS at room temperature to block nonspecific binding sites. After removal of the blocking solution, samples were incubated for 16 h at 4 °C in a humidified chamber with one of the following primary antibodies: anti-porcine CD172a-fluorescein isothiocyanate (FITC) SWC3 IgG1 (Southern Biotech), anti-porcine CD11R1-unlabeled mouse IgG1 (AbD Serotec, Kidlington, Oxford, United Kingdom), anti-porcine major histocompatibility complex class II (MHC-II)-unlabeled mouse IgG2a, anti-porcine TLR2-unlabeled rabbit IgG (Santa Cruz); or anti-porcine TLR4-unlabeled rabbit IgG (Santa Cruz). Samples were washed in PBS and then treated with one of the following secondary antibodies: anti-mouse IgG2a-FITC (AbD Serotec), anti-mouse IgG-FITC (AbD Serotec), or anti-rabbit IgG-FITC (Santa Cruz), when necessary.

### Electron microscopy

Morphology of porcine PPMPs and MoDCs, and phagocytosis of lactobacilli by porcine APCs was examined by Transmission Electron Microscopy (TEM) or Scanning Electron Microscopy (SEM). PPMPs or MoDCs cells were seeded on 12-well type I collagen coated coverglass (IWAKI, Tokyo, Japan). For SEM studies, porcine APCs were washed with 0.1 M HEPES buffer and fixed in 2.5 % glutaraldehyde for 2 h at room temperature. Then, cells were washed with PBS, and replacement with alcohol, freeze dehydration and platinum spraying were conducted. Finally, samples were evaluated with SEM (SU8000 Type II, HITACHI, Tokyo, Japan). For TEM studies, porcine APCs were fixed with 2.5 % glutaraldehyde in 0.1 M phosphate buffer (pH 7.4, 4 °C) for 2 h, and then treated with 1 % osmium in 0.1 M phosphate buffer (pH 7.4, 4 °C) for 4 h. These samples were embedded in epoxy resin according to the routine method. Ultrathin sections were double-stained with platinum blue diluted 10 times with distilled water (TI-blue staining kit: Nisshin, Tokyo, Japan), and a lead solution for 10 min each at room temperature. Samples were evaluated with TEM (H-7650, Hitachi, Tokyo, Japan).

### Flow cytometric analysis

Flow cytometry was used to characterize CD172a^+^CD11R1^+^, and CD172a^−^CD11R1^+^ cells within the MHC-II^+^ population of porcine PPMPs and MoDCs. Cells were isolated as described above and incubated with the following primary antibodies: anti-porcine CD172a-PE SWC3 IgG1 (Southern Biotech), anti-porcine CD11R1-unlabeled IgG1 (AbD Serotec), and anti-porcine MHC-II-unlabeled IgG2a (VMRD). Unlabeled monoclonal antibodies were detected by the following secondary antibodies: anti-mouse IgG2a-FITC (AbD Serotec), and anti-mouse IgG1-PerCP/Cy5.5 (Bio Legend, San Diego, CA, USA). Isotype controls were obtained by incubating cells with irrelevant mouse IgG1-PE; IgG-FITC; or IgG1-PerCP antibodies (eBioscience, San Diego, CA, USA). Analysis was performed with FACS-Calibur™ (BD, Franklin Lakes, NJ), which was equipped with Cell-Quest software. Data analysis was performed using the FlowJo software (Tree Star, Ashland, OR) as described before [15].

Phagocytosis of lactobacilli by porcine PPMPs and MoDCs was also evaluated by flow cytometry. Cultures of *L. jensenii* TL2937 and *L. plantrum* TL2766 were grown in MRS as described before. Then, lactobacilli were washed and suspended in PBS. Each lactobacilli strain (10^11^ cfu) were incubated with 3 mg cFDA in 1 mL PBS for 1 h at 37 °C in a water bath, then washed twice with PBS and suspended in 1 ml PBS. These bacterial samples were heat-treated at 56 °C for 30 min and stored at −80 °C until use.

### Quantitative expression analysis using real-time PCR

Two-step real-time quantitative PCR was performed to evaluate the expression of mRNAs in porcine PPMPs or MoDCs [17, 21]. Total RNA was obtained from samples using TRIzol (Sigma). All cDNAs were synthesized using a Quantitect Reverse Transcription kit (Qiagen, Tokyo, Japan) according to the instructions of the manufacturer. Real-time quantitative PCR was performed with a 7300 Real-time PCR System (Applied Biosystems, Warrington, UK) and Platinum SYBR Green qPCR SuperMix UDG with ROX (Invitrogen). The primers used for the analysis of IL-1β, IL-12p40 and IL-10 were described previously [15, 22]. PCR cycling conditions were 2 min at 50 °C, 2 min at 95 °C, and then 40 cycles of 15 s at 95 °C, 30 s at 60 °C, and 30 s at 72 °C. The reaction mixtures contained 5 μl of the sample cDNA and 15 μl of the master mix, including appropriate sense and antisense primers. β-actin expression was used as an internal control to normalize differences between samples, and therefore mRNA levels of β-actin were determined in each sample. A relative index was calculated after normalization with β-actin and results were expressed as normalized fold expression based on non-stimulated cell controls set as 1.0.

### Statistical analysis

Statistical analyses were performed using GLM and REG procedures (SAS computer program, 1994). Comparisons between mean values were carried out using one-way ANOVA and Fisher’s least significant difference (LSD) test. For these analyses, *p*-values < 0.05 were considered significant.

## Results

### Lactobacillus jensenii TL2937 is efficiently phagocytosed by porcine adherent PPMPs

**Fig. 1:**
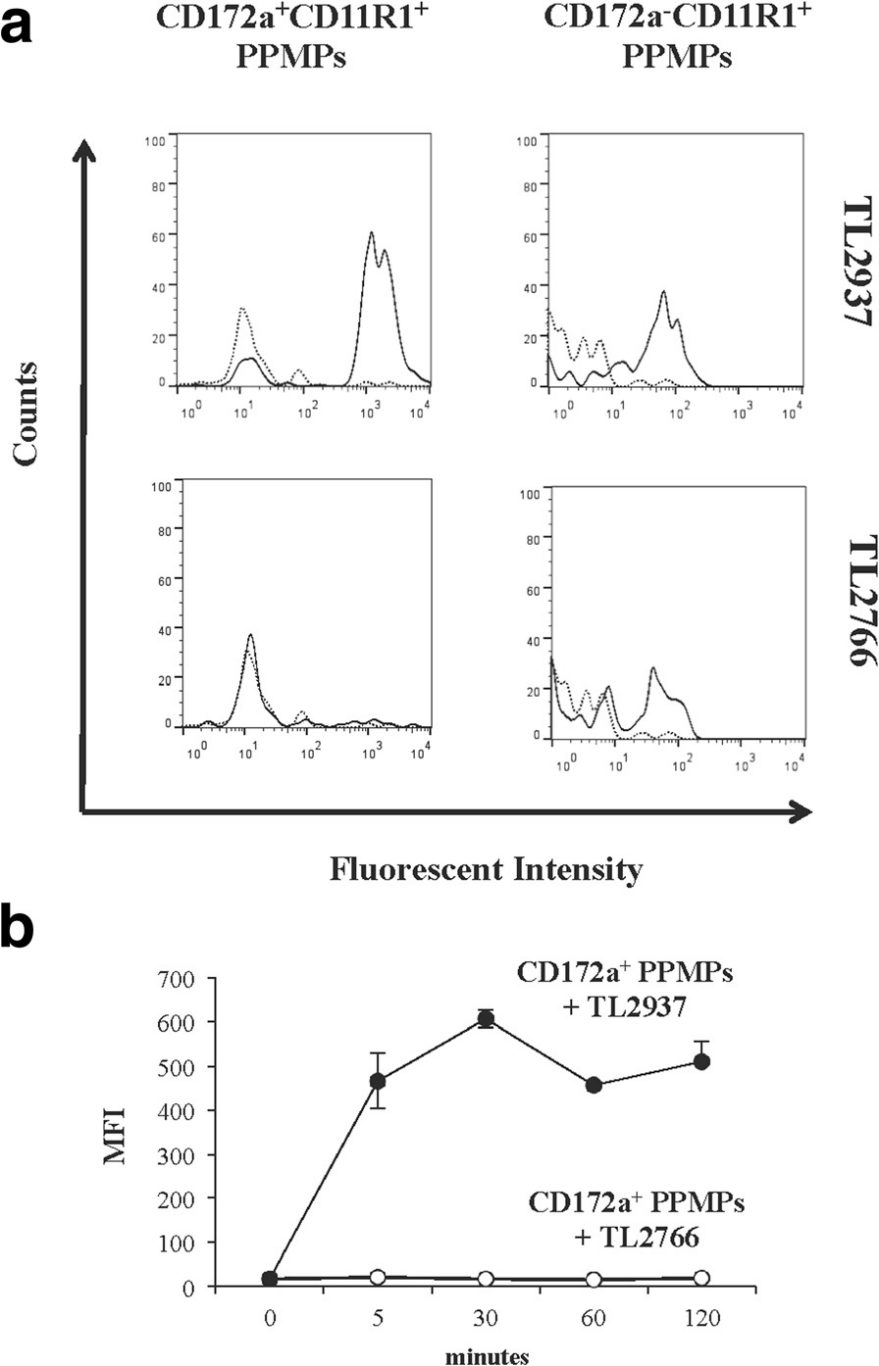
Phagocytosis of immunobiotic *Lactobacillus jensenii* TL2937 by mononuclear phagocytes from porcine Peyer’s patches (PPMPs). Adherent PPMPs were treated with the immunobiotic strain *L. jensenii* TL2937 or the non-immunoregulatory strain *L. plantarum* TL2766. Untreated adherent PPMPs were used as controls. Bacterial phagocytosis by adherent CD172a^+^CD11R1^+^ or CD172a^−^CD11R1^+^ PPMPs was evaluated using flow cytometric analysis. Histograms represent data from the flow cytometric analysis as follows: cells treated with lactobacilli (solid line), and untreated control cells (dot line) (**a**). Isolated CD172a^+^ PPMPs cells were obtained by a MACS cell separation system (magnetic cell labeling). Isolated CD172a^+^ cells were treated with *L. jensenii* TL2937 or *L. plantarum* TL2766. Bacterial phagocytosis was evaluated using flow cytometric analysis (**b**). In all the experiments cells were used in a concentration of 5.0 × 10^6^ cells/well. Values of mean fluorescence intensity (MFI) are shown for each group. The results represent data from three independent experiments using different donors

The expression of CD172a and CD11R1 were analyzed in MHC-II^+^ cells from porcine adherent PPMPs to define two APCs populations: CD172a^+^CD11R1^+^MHC-II^+^, and CD172a^−^CD11R1^+^MHC-II^+^ cells as described previously [15]. We evaluated the capacity of both types of adherent PPMPs to phagocytose the immunobiotic strain *L. jensenii* TL2937 and the negative control *L. plantarum* TL2766 by using flow cytometry (Fig. 1a). CD172a^+^CD11R1^+^ adherent PPMPs efficiently phagocytosed the TL2937 strain while CD172a^−^CD11R1^+^ adherent PPMPs showed a lower capacity to phagocytose this bacterium. In addition, CD172a^−^CD11R1^+^ adherent PPMPs phagocytosed the TL2766 strain while no phagocytic activity was observed in CD172a^+^CD11R1^+^ adherent PPMPs for this bacterium. These results indicated that adherent CD172a^+^CD11R1^+^ PPMPs differentially recognized both lactobacilli. Therefore, the following experiments were focused in this cell population. CD172a^+^ cells were isolated from porcine PPs using a magnetically activated cell sorting method, and phagocytosis was analyzed again by flow cytometry (Fig. 1b). Results confirmed the high capacity of isolated CD172a^+^ PPMPs to phagocytose *L. jensenii* TL2937 and the lower phagocytosis of *L. plantarum* TL2766 (Fig. 1b, c).

**Fig. 2:**
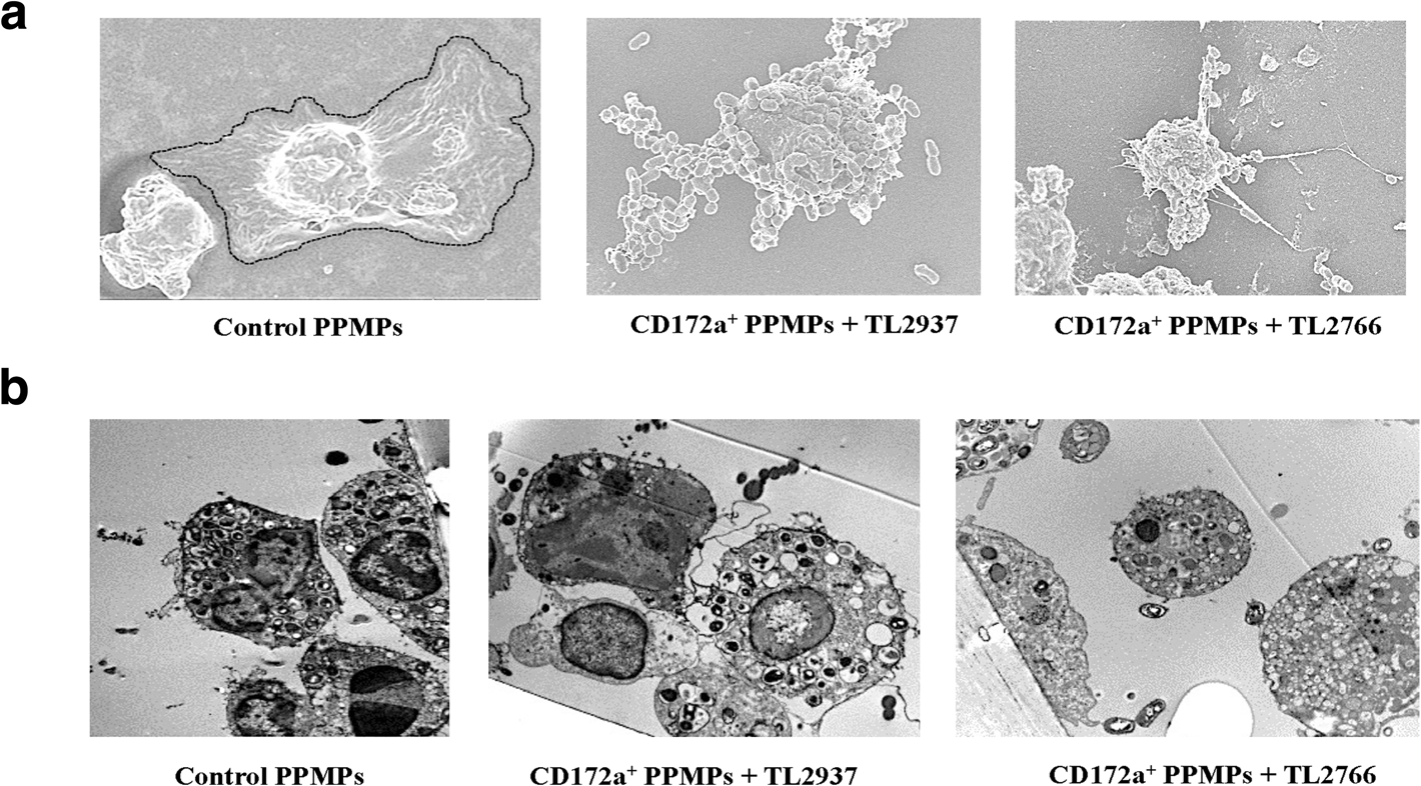
Phagocytosis of immunobiotic *Lactobacillus jensenii* TL2937 by mononuclear phagocytes from porcine Peyer’s patches (PPMPs). Isolated CD172a^+^ PPMPs cells were obtained by a MACS cell separation system (magnetic cell labeling). Isolated CD172a^+^ PPMPs were treated with the immunobiotic strain *L. jensenii* TL2937 or the non-immunoregulatory strain *L. plantarum* TL2766. Untreated CD172a^+^ PPMPs were used as controls. Bacterial phagocytosis by PPMPs was evaluated using (**a**) Scanning Electron Microscopy (SEM) or (**b**) Transmission Electron Microscopy (TEM). In all the experiments cells were used in a concentration of 1.0 × 10^7^ cells/well. Photos represent data from three independent experiments using different donors

In addition, transmission and scanning electron microscopies were performed to evaluate lactobacilli and porcine isolated CD172a^+^ PPMPs interactions. As shown in Fig. 2, when analyzing co-cultures of APCs and *L. jensenii* TL2937, a high number of bacterial cells could be found attached to isolated CD172a^+^ PPMPs and within the cells. On the contrary, low numbers of bacterial cells were found attached or in the cytoplasm of isolated CD172a^+^ PPMPs stimulated with *L. plantarum* TL2766 (Fig. 2).

**Fig. 3:**
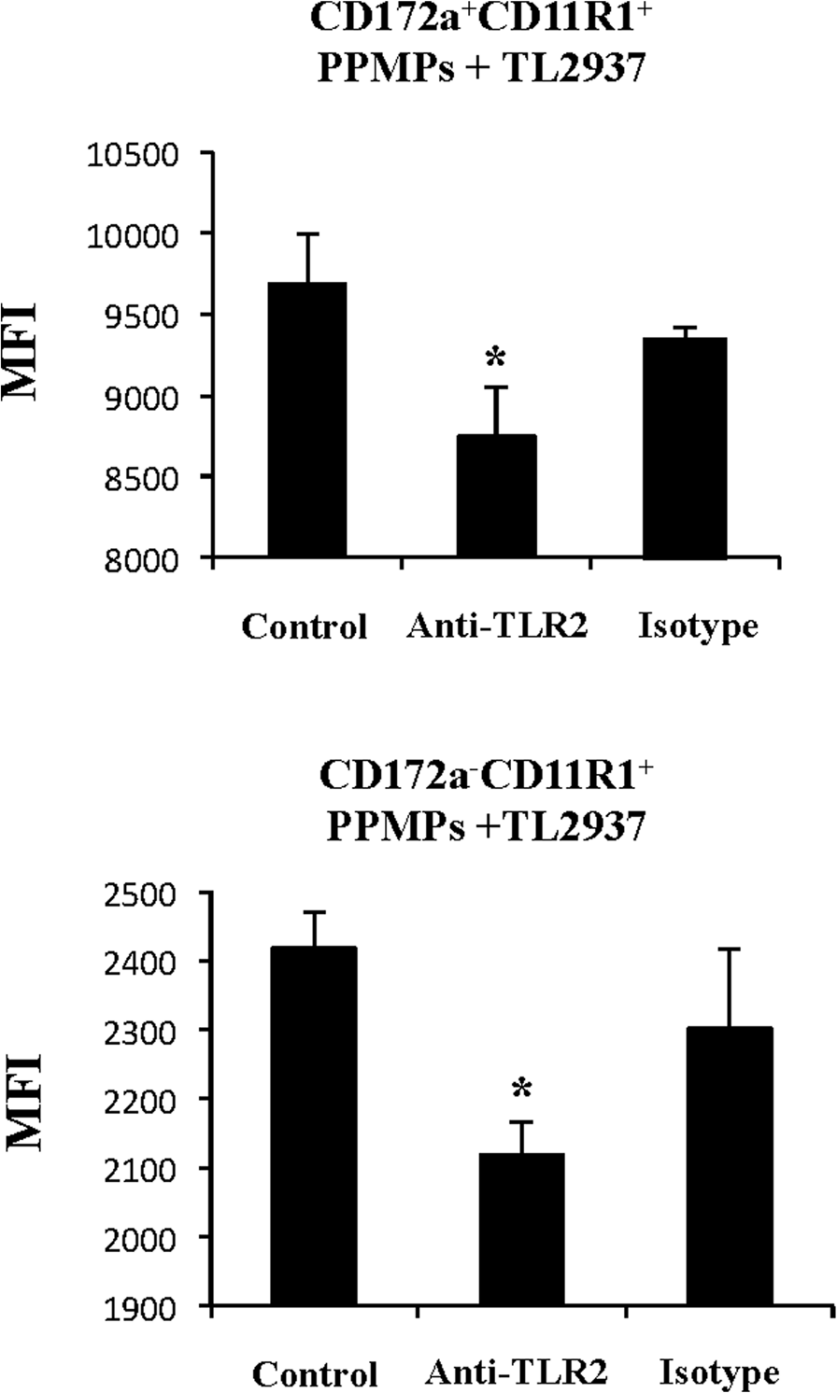
Role of TLR2 in phagocytosis of immunobiotic *Lactobacillus jensenii* TL2937 by mononuclear phagocytes from porcine Peyer’s patches (PPMPs). Adherent PPMPs were treated with the immunobiotic strain *L. jensenii* TL2937 in the presence (anti-TLR2 group) of absence (control group) of blocking anti-TLR2 antibodies. Adherent PPMPs treated with isotype antibodies were used as controls (isotype group). Bacterial phagocytosis by adherent CD172a^+^CD11R1^+^ or CD172a^−^CD11R1^+^ PPMPs was evaluated using flow cytometric analysis. In experiments cells were used in a concentration of 5.0 × 10^6^ cells/well. Values of mean fluorescence intensity (MFI) are shown for each group. The results represent data from three independent experiments using different donors

We previously evaluated the role of TLR2 in the immunomodulatory effect of *L. jensenii* TL2937 by performing comparative studies with the TLR2 agonist Pam3CSK4 and with anti-TLR2 antibodies. We demonstrated that TLR2 is involved in the induction of IL-1β, IFN-γ, and IL-10 in porcine APCs [15]. Then, we next aimed to evaluate whether TLR2 has a role in the differential phagocytic activity of porcine adherent PPMPs. Adherent cells were stimulated with *L. jensenii* TL2937 or *L. plantarum* TL2766 in the absence of presence of anti-TLR2 antibodies (Fig. 3). Blocking antibodies reduced the values of MFI in both CD172a^+^CD11R1^+^ and CD172a^−^CD11R1^+^ adherent PPMPs stimulated with the immunobiotic strain *L. jensenii* TL2937, while no effect was observed for *L. plantarum* TL2766 (data not shown).

### Lactobacillus jensenii TL2937 is efficiently phagocyted by porcine MoDCs

**Fig. 4:**
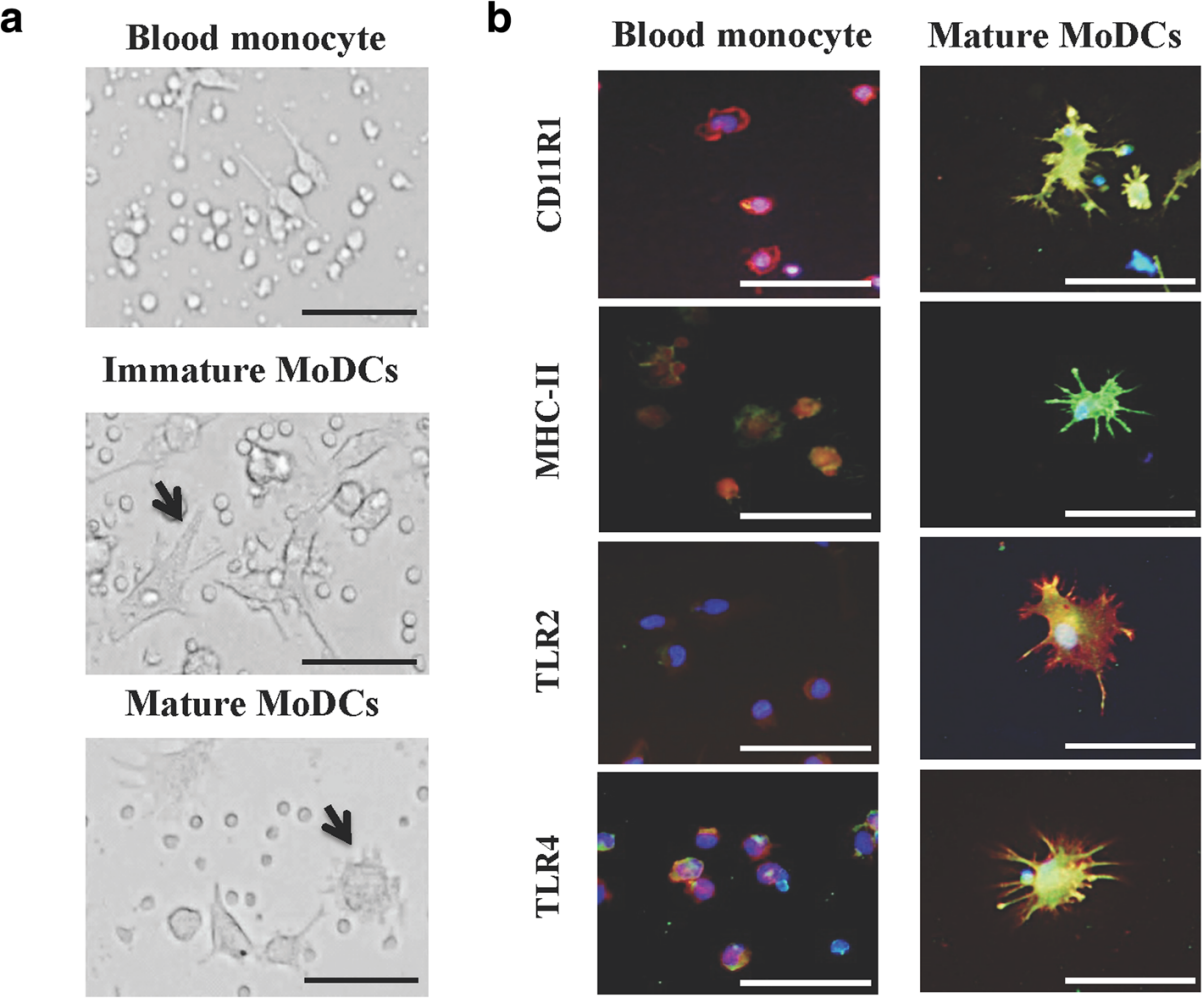
Generation of porcine blood monocytes-derived dendritic cells (MoDCs). Porcine immature MoDCs were generated from blood monocytes after the differentiation with GM-CSF and IL-4 for 5 days. Maturation of MoDCs was induced by stimulation with LPS. Cell morphology was evaluated by microscopic analysis (**a**). Expression of CD11R1, MHC-II, TLR2, and TLR4 in blood monocytes and mature MoDCs was determined by fluorescent microscopy (**b**). Photos represent data from three independent experiments using different donors. In experiments cells were used in a concentration of 5.0 × 10^6^ cells/well. Scale bar = 50 μm

Porcine immature MoDCs were generated from blood monocytes after the differentiation with GM-CSF and IL-4 for 5 days. Maturation of MoDCs was induced by stimulation with LPS. Morphologically, MoDCs displayed a significant size increase, irregular shape when compared with blood monocytes (Fig. 4a). Moreover, MoDCs developed irregular pseudopodia on the cell surface with this differentiation protocol, which was more evident in mature MoDCs. In addition, MoDCs showed a higher expression of CD11R1, MHC-II, TLR2, and TLR4 expression when compared to blood monocytes as determined by fluorescent microscopy (Fig. 4b).

**Fig. 5:**
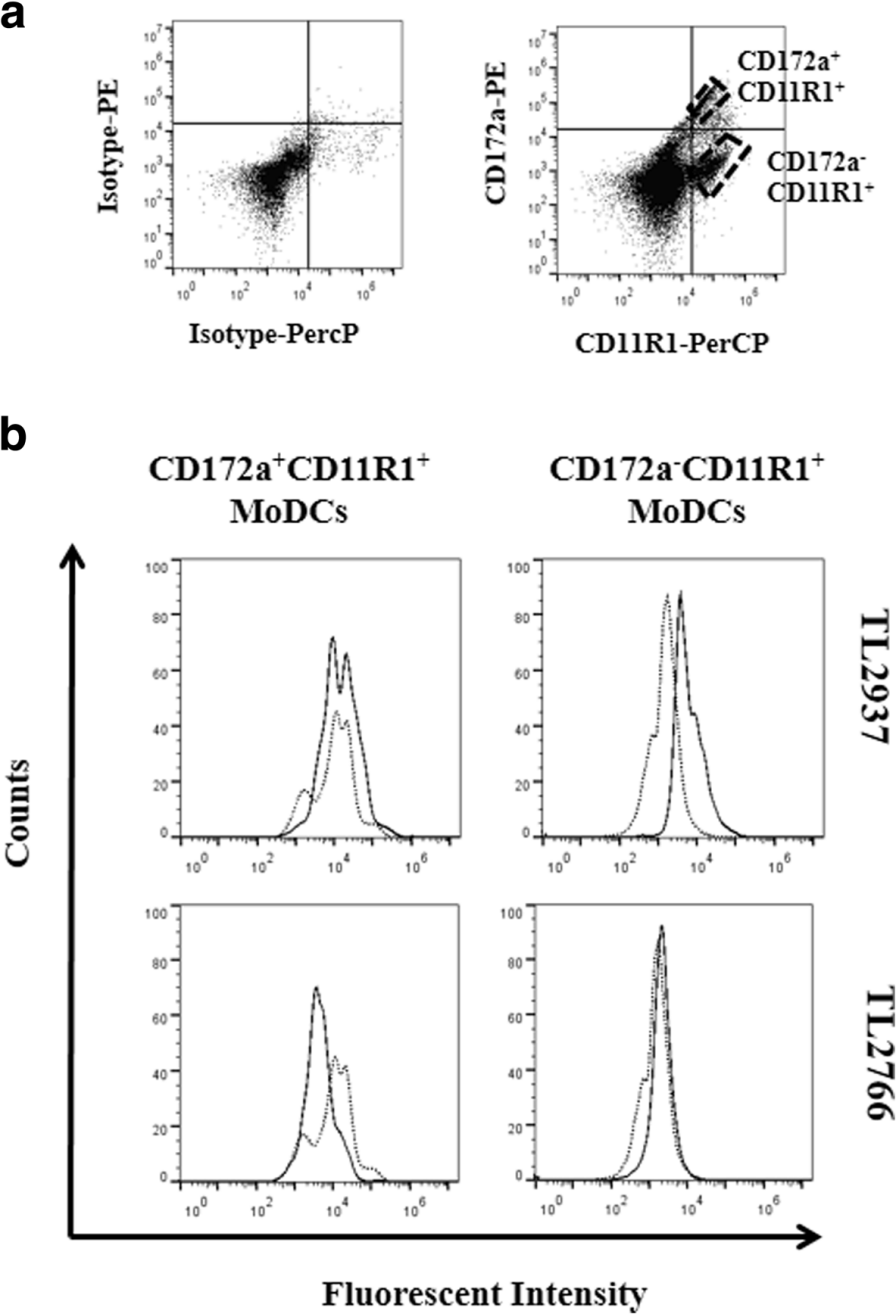
Phagocytosis of immunobiotic *Lactobacillus jensenii* TL2937 by porcine blood monocytes-derived dendritic cells (MoDCs). MoDCs were stained with antibodies for CD172a, and CD11R1 and two cell populations were defined: CD172a^+^CD11R1^+^ or CD172a^−^CD11R1^+^ (**a**). MoDCs were treated with the immunobiotic strain *L. jensenii* TL2937 or the non-immunoregulatory strain *L. plantarum* TL2766. Untreated MoDCs cells were used as controls. Bacterial phagocytosis by CD172a^+^CD11R1^+^ or CD172a^−^CD11R1^+^ MoDCs cells was evaluated using flow cytometric analysis (**b**). Histograms represent data from the flow cytometric analysis, as follows: cells treated with lactobacilli (solid line) and, untreated control cells (dot line). The results represent data from three independent experiments using different donors. In experiments cells were used in a concentration of 5.0 × 10^6^ cells/well

**Fig. 6:**
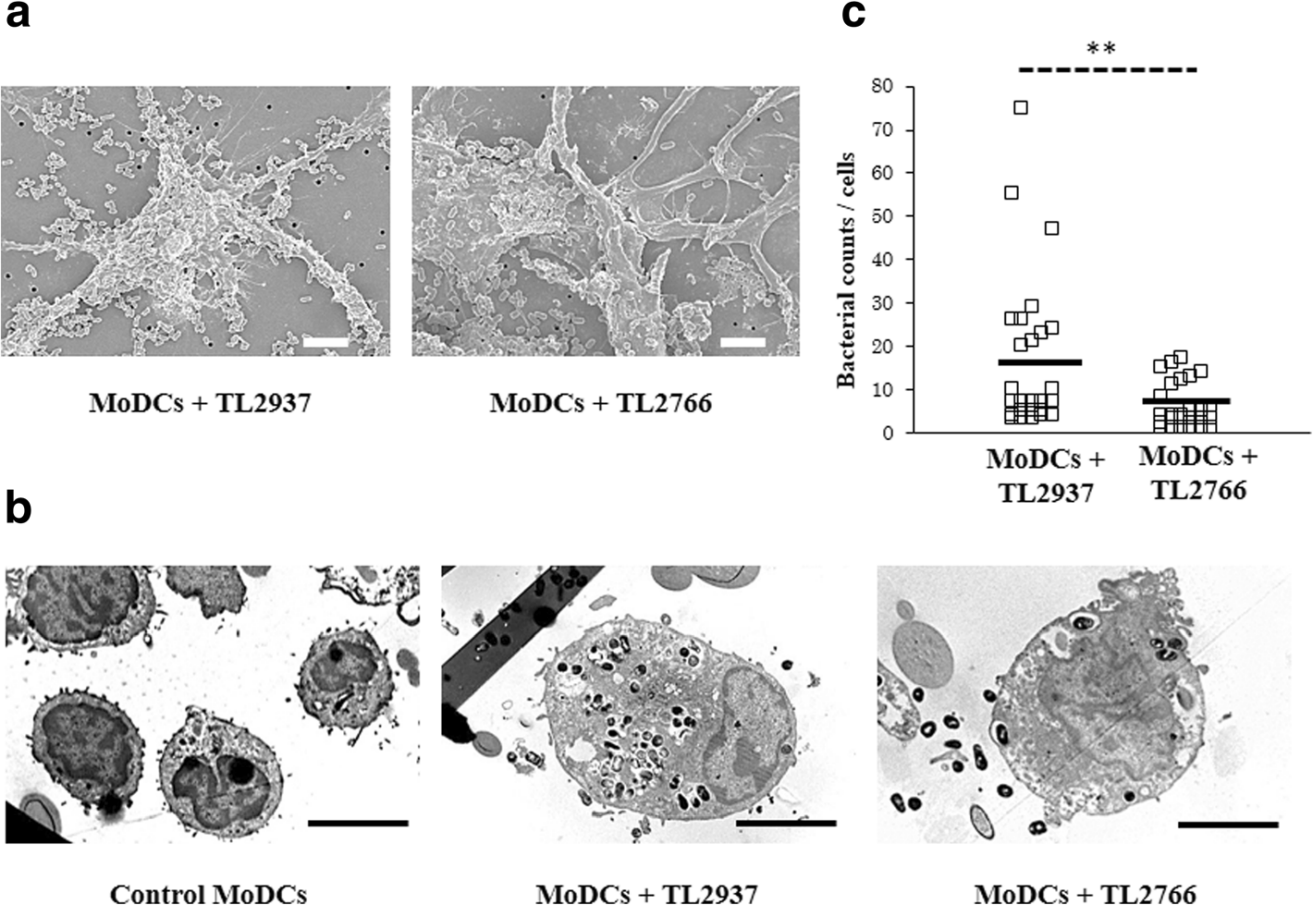
Phagocytosis of immunobiotic *Lactobacillus jensenii* TL2937 by porcine blood monocytes-derived dendritic cells (MoDCs). MoDCs were treated with the immunobiotic strain *L. jensenii* TL2937 or the non-immunoregulatory strain *L. plantarum* TL2766. Untreated MoDCs were used as controls. Bacterial phagocytosis by MoDCs was evaluated using (**a**) Scanning Electron Microscopy (SEM) or (**b**) Transmission Electron Microscopy (TEM). The number of bacteria incorporated in the cells was counted using TEM and results were expressed as the average of bacteria number per cell (**c**). Photos represent data from three independent experiments using different donors. In experiments cells were used in a concentration of 1.0 × 10^7^ cells/well. Scale bar = 50 μm

Within porcine immature MoDCs two populations of DCs could be defined by using CD172a and CD11R1 markers as shown in Fig. 5a: CD172a^+^CD11R1^+^ and CD172a^−^CD11R1^+^ MoDCs. We also evaluated the capacity of these two types of immature MoDCs to phagocytose *L. jensenii* TL2937 and *L. plantarum* TL2766 by using flow cytometry and fluorescent microscopy (Fig. 5). Similarly, to the results obtained with PPMPs, the TL2937 strain was more efficiently phagocyted by CD172a^+^CD11R1^+^ and CD172a^−^CD11R1^+^ MoDCs than *L. plantarum* TL2766. Electron microscopy studies confirmed those findings. As shown in Fig. 6, when analyzing co-cultures of MoDCs and *L. jensenii* TL2937, a high number of bacterial cells could be found attached to and within the cells. On the contrary, low numbers of bacterial cells were found attached or in the cytoplasm of MoDCs stimulated with *L. plantarum* TL2766.

### Lactobacillus jensenii TL2937 functionally modulates porcine immature MoDCs

**Fig. 7:**
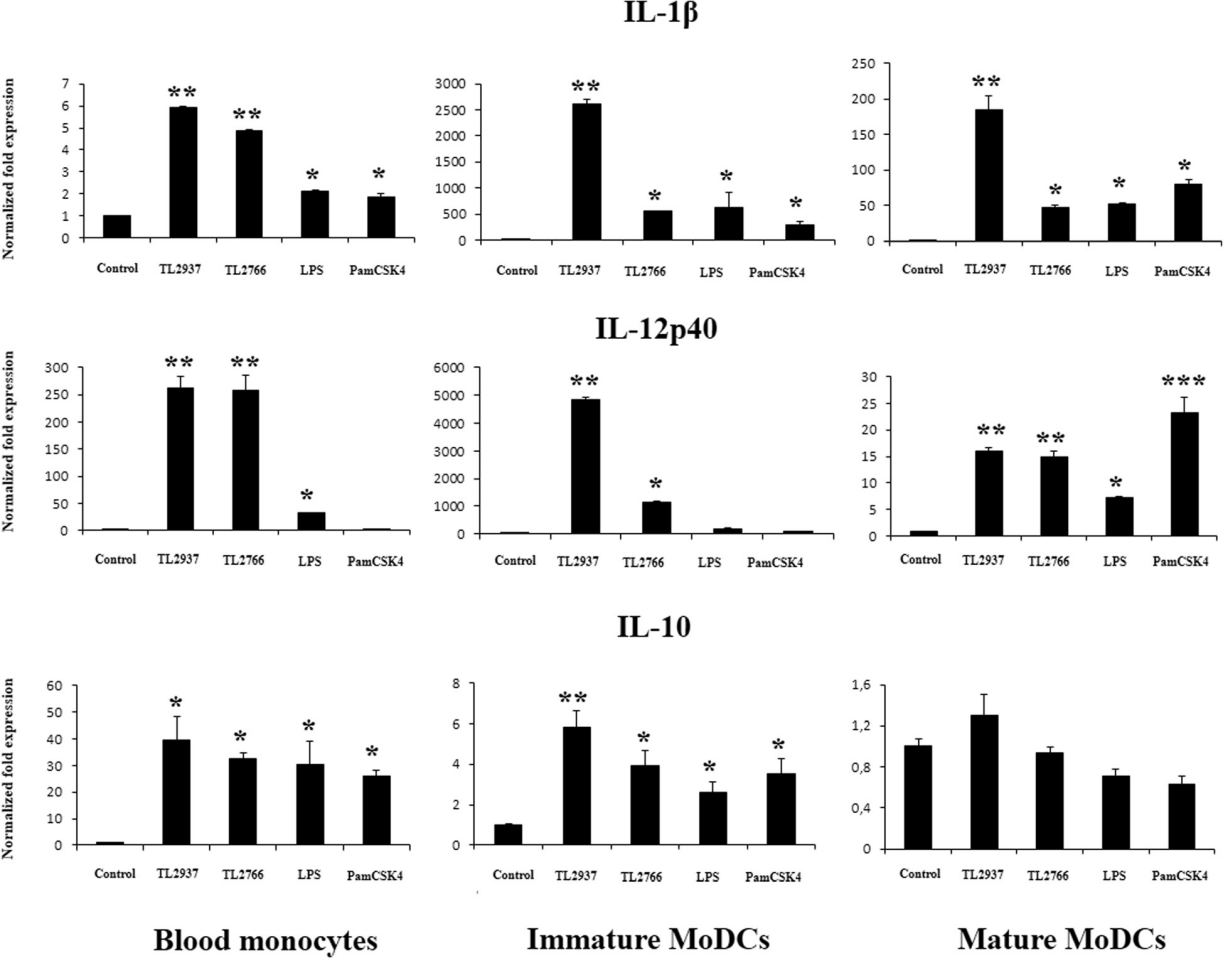
Blood phagocytes cytokine response to immunobiotic *Lactobacillus jensenii* TL2937 stimulation. Porcine blood monocytes and, immature and mature monocytes-derived dendritic cells (MoDCs) were treated with the immunobiotic strain *L. jensenii* TL2937 or the non-immunoregulatory strain *L. plantarum* TL2766. Untreated MoDCs cells were used as negative controls. Cell treated with LPS or PamC3SK4 were used as positive controls. Expression of IL-1β, IL-12p40, and IL-10 mRNAs was examined using RT-PCR. In experiments cells were used in a concentration of 5.0 × 10^6^ cells/well. The results represent data from three independent experiments using different donors. Asterisks indicate significant differences * (*P* < 0.05), ** (*P* < 0.01)

Finally, we aimed to evaluate whether the immunobiotic strain TL2937 was able to functionally modulate porcine blood APCs. For this purpose, blood monocytes and immature and mature MoDCs were stimulated with *L. jensenii* TL2937 and the expression of IL-1β, IL-12p40, and IL-10 were evaluated by RT-PCR (Fig. 7). *L. plantarum* TL2766 were used as negative control, and LPS and Pam3CSK4 as positive controls.

*L. jensenii* TL2937 and *L. plantarum* TL2766 were equally efficient to improve the production of IL-1β and IL-12 in blood monocytes while cells stimulated with the TLR2 or TLR4 agonists showed lower levels of both cytokines when compared to lactobacilli-treated cells. On the other hand, IL-10 was improved in blood monocytes by all treatments without significant differences between them (Fig. 7). In addition, all the treatments increased the expression of IL-1β, IL-12 and IL-10 in immature MoDCs, however, levels of the three cytokines were significantly higher in TL2937-treated cells when compared with the other groups (Fig. 7). No effect on IL-10 production was observed in mature MoDCs stimulated with lactobacilli or TLRs agonists. All the treatments increased the expression of IL-1β and IL-12 in mature MoDCs, however, *L. jensenii* TL2937 was more effective to improve IL-12 levels (Fig. 7).

## Discussion

Several *Lactobacillus* strains have well-documented immunobiotic functions since it was demonstrated that they confer immune health-promoting effects in humans or animals. Among them, the immunobiotic strain *L. jensenii* TL2937 has been shown to improve immune health in pigs [9, 16]. Our previous work demonstrated that stimulation of porcine PPMPs with *L. jensenii* TL2937 induced phenotypical and functional changes in these APCs in the absence of inflammatory stimuli. In fact, the treatment of PPMPs with *L. jensenii* TL2937 upregulated MHC-II and CD80/86 molecules and increased their expression of IL-10, IL-1β, and IFN-γ [15]. We also reported that pretreatment of PPMPs with the immunobiotic TL2937 strain resulted in differential response to LPS challenge, indicating that this bacterium is capable of modulate TLR4-mediated inflammatory response in PPMPs in a beneficial way [15]. Moreover, our results indicated that TLR2 has an important role in the immunoregulatory effect of *L. jensenii* TL2937, as demonstrated by comparative experiments using anti-TLR2 antibodies, and the TLR2 agonist Pam3CSK4 [15].

One key characteristic of APCs in their capacity to induce and modulate immune responses is their ample ability to acquire exogenous antigens by different endocytic processes. APCs take microorganisms through a variety of mechanisms, including uptake by constitutive macropinocytosis and via receptor-mediated endocytosis and phagocytosis [1]. To gain a better insight into the immunobiotic properties of *L. jensenii* TL2937, we investigated here the role of phagocytosis and TLR2 in the molecular interaction between this strain and porcine APCs. We showed a high ability of porcine CD172a^+^ PPMPs to phagocytose *L. jensenii* TL2937. Interestingly, our results also revealed a reduced capacity of the non-immunomodulatory *L. plantarum* TL2766 to be phagocytosed by porcine CD172a^+^ PPMPs with a diminished bacterial uptake. Therefore, considering that phagosomes are important for the initiation and amplification of immune signaling pathways [3, 4]; our results indicate that the immunobiotic properties of *L. jensenii* TL2937 would be related to its higher capacity to interact with PPMPs and be phagocytosed.

The need of an efficient phagocytosis for the induction of immunomodulatory activities by beneficial bacteria in APCs has been reported for the immunobiotic GG strain. Pili, which are encoded by the spaCBA gene cluster, are expressed at the cell surface of the probiotic *L. rhamnosus* GG [23], and it was demonstrated that the SpaC subunit is the responsible for the high mucus-binding capacity [24]. In fact, it was recently reported that SpaCBA pili of GG strain are crucial for its capacity to adhere to murine macrophages, and that this interaction stimulates bacterial phagocytosis [25]. SpaCBA pili promote bacterial uptake partially through CR3 (CD11b/CD18) that is a receptor present on most immune cells, including monocytes, macrophages, and DCs. Moreover, authors observed that the pilus-mediated enhanced bacteria-host cell interaction was related to the immunoregulatory ability of the GG strain. Interestingly, the work speculates that the pili do not directly modulate the production of cytokines, but rather mediate close interaction of lactobacilli with macrophages, resulting in an improved delivery of immunomodulatory molecules [25]. Then, different receptors would be involved in phagocytosis and cytokine induction in *L. rhamnosus* GG-treated macrophages.

Our results showed that interaction of *L. jensenii* TL2937 with porcine APCs would be partially dependent on TLR2 [15]. Therefore, there are two possibilities to explain the role of TLR2 in the immunomodulatory effect of the TL2937 strain: a) similarly to CR3 for *L. rhamnosus* GG, TLR2 would mediate a close interaction of *L. jensenii* TL2937 with porcine APCs resulting in an enhanced contact of immunomodulatory molecules that activate other PPRs or; b) TLR2 would be involved in both close contact/phagocytosis and signaling/cytokine induction in *L. jensenii* TL2937-treated porcine APCs together with other PRRs. What it is clear from our studies is that TLR2 is not the only receptor involved in the effect of the immunobiotic *L. jensenii* TL2937. The majority of the studies evaluating the interactions between immunobiotic microorganism and PRRs were limited to interactions between a single ligand and its corresponding receptor. However, microorganisms such as immunobiotic bacteria express more than one PRRs ligand and their interactions with immune cells are more complex and probably involve more than one PRRs. Therefore, further studies are needed to identify the exact role of TLR2 in the immunomodulatory activity of *L. jensenii* TL2937 strain as well as the role of other PPRs.

It is well known that each of the several DCs subpopulations has distinct phenotypic and functional properties. MoDCs are a type of highly plastic APCs that are able to acquire a multitude of functional capabilities, some of which are shared with resident mucosal DCs [26]. Mouse and human MoDCs have been widely used for the study of immune responses in DCs including the immunomodulatory effects pathogens and beneficial bacteria. More recently, porcine MoDCs have been used with the same purpose. Studies have demonstrated that porcine MoDCs are able to respond to the challenge with different TLRs agonists [27], and that these cells are a useful *in vitro* tool for studying immune responses against porcine pathogens [28, 29] as well as for the evaluation of the immunomodulatory effects of feed dietary supplements [30].

In this work, we demonstrated that *L. jensenii* TL2937 was able to modulate porcine MoDCs and that its effects were similar to those observed in PPMPs. We found that *L. jensenii* TL2937 strain was able to improve the expression of IL-1β, IL-12 and IL-10 in immature MoDCs that resembles the effect of this immunobiotic *Lactobacillus* in PPMPs [15]. Moreover, similarly to PPMPs the immunomodulatory effects were related to the higher capacity of *L. jensenii* TL2937 to be phagocyted by immature MoDCs. On the other hand, *L. jensenii* TL2937 was not able to increase the expression of IL-10 in porcine mature MoDCs. In addition, the TL2937 strain improved the levels of IL-1β but in a similar way of the negative control strain TL2766. Those results indicated that porcine mature MoDCs are not able to detect differences between both lactobacilli. Most DCs exist in a quiescent immature state and they can be activated to a mature state by a wide variety of stimulatory ligands including MAMPs. Whereas immature DCs have a high phagocytic capacity, DCs lose this ability upon maturation [5]. In addition, it was observed that DCs are able to induce tolerance *in vivo* when they are not fully mature as in the steady state [31]. Those differences between immature and mature MoDCs would explain the distinct response of both populations to *L. jensenii* TL2937 stimulation. Furthermore, those characteristics of porcine immature MoDCs would allow us to speculate that these cells are better *in vitro* tools to study the immunomodulatory effects of immunobiotic bacteria.

## Conclusion

Microbial recognition by APCs constitutes a major step in the initiation of immune mechanisms that lead to the development of resistance against infectious agents or tolerance against commensal bacteria. These recognition events are primarily mediated through MAMPs-PRRs interactions that mediate phagocytosis and signaling. A challenge for the coming years will be advance in the full understanding of these molecular interactions between immunobiotic bacteria and porcine immune cells. Those studies will be crucial for the successful development of functional feeds for the porcine host. This study is a step forward in that direction.

## Abbreviations

APCs, antigen presenting cells; DCs, dendritic cells; IL, interleukin; IRAK-M, interleukin-1 receptor-associated kinase M; LPS, lipopolysaccharide; MAMPs, microbial-associated molecular patterns; MoDCs, monocyte-derived dendritic cells; PPMPs, mononuclear phagocytes from porcine Peyer’s patches; PPs, Peyer’s patches; PRRs, pattern recognition receptors; SIGIRR, single immunoglobulin IL-1-related receptor; TLRs, Toll-like receptors; TSLP, thymic stromal lymphopoietin
